# Dr. Thomas McCann

**Published:** 1903-07

**Authors:** 


Dr. Thomas McCann, of Pittsburg, died May 9, 1903, after a prolonged illness, aged 40 years. He received his medical degree at Bellevue Hospital Medical College in 1886, after which he served as interne at Bellevue hospital. When his term expired as resident physician he returned to Pittsburg and commenced practice with his father, Dr. James McCann, one of the leading surgeons of that city. The younger McCann developed surgical talent early in his professional career and became one the leaders of that branch in Pittsburg. He was a member of several local societies, and in 1898 was elected Fellow of the American Association of Obstetricians and Gynecologists. He was professor of the practice of surgery and clinical surgery in the Western Pennsylvania Medical College, and consulting surgeon to Saint John's Hospital, Allegheny.

